# GPX Knockdown Is Associated with Altered Redox Homeostasis, Plant Development, and DNA Methylation-Related Profiles in Rice

**DOI:** 10.3390/epigenomes10030049

**Published:** 2026-07-24

**Authors:** Pedro Alexander Velasquez-Vasconez, Marina de Lima Nogueira, Carlos Betancourth García, Claudia Elizabeth Salazar-González, Angie Fernanda Riascos-España

**Affiliations:** 1 Department of Production and Plant Protection, University of Nariño, Pasto 520001, Colombia; 2 School of Basic Sciences, Technology and Engineering, National Open and Distance University, Pasto 520001, Colombia; 3 Department of Biological and Health Sciences, Federal University of Amapá, Macapá 68903-419, AP, Brazil; 4 Faculty of Exact and Natural Sciences/Biology Program, University of Nariño, Pasto 520001, Colombia; riascosfer0824@gmail.com

**Keywords:** glutathione peroxidase, oxidative stress, DNA methylation, redox homeostasis, epigenetic plasticity, *Oryza sativa*

## Abstract

Background: Glutathione peroxidases (GPXs) regulate peroxide detoxification and redox signaling, but their relationship with DNA methylation remains unclear in Oryza sativa. This study evaluated whether silencing mitochondrial GPX1 and GPX3 is associated with changes in growth, antioxidant activity, and DNA methylation-related profiles. Methods: Non-transformed plants (NT) and five GPX-silenced lines were evaluated in a randomized complete block design. Morphophysiological traits, antioxidant enzyme activities, total 5-methylcytosine content, and methylation-sensitive restriction profiles were analyzed. Results: GPX silencing impaired early establishment and significantly affected flowering time and leaf, root, seed, and total biomass. Total dry biomass decreased by 62.1% in the most affected GPX1 lines and by 29.2% in GPX3 lines relative to NT plants. Root biomass declined by up to 86.1%, and flowering was delayed by up to 30.7 days. Genotype significantly affected GPX-associated and glutathione reductase activities, whereas no significant genotype effects were detected for catalase, ascorbate peroxidase, or superoxide dismutase activities. GPX-associated and glutathione reductase activities were strongly correlated (r = 0.85, *p* < 0.001), consistent with selective alteration of GPX-associated and glutathione-linked redox metabolism. Total 5-methylcytosine content decreased by 41–42% in GPX-silenced groups. However, increased McrBC digestion and unchanged HpaII/MspI profiles indicated that methylation-related changes were nonuniform and depended on the genomic sites recognized by each enzymatic assay. Conclusions: These findings show that mitochondrial GPX knockdown is associated with impaired rice growth and reproductive development, as well as with altered total 5-methylcytosine content and restriction-sensitive methylation profiles, suggesting a potential relationship among redox homeostasis, developmental regulation, and epigenetic plasticity that requires further validation using locus-resolved and mechanistic approaches.

## 1. Introduction

Reactive oxygen species (ROS) are now understood less as indiscriminate by-products of metabolism than as compartmentalized regulators whose effects depend on dose, timing, and subcellular origin [1]. In plants, this principle is especially important for mitochondria, where electron transport, energy status, and redox buffering are tightly coupled to developmental decisions and stress acclimation [2]. Recent syntheses of thiol-redox regulation emphasize that the glutathione/glutaredoxin system is not merely a detoxification buffer but a signaling platform that links peroxide processing to protein thiol control, glutathionylation, and metabolic homeostasis [3,4]. Likewise, current organelle-signaling frameworks have reinforced the broader concept that organellar redox status can be translated into nuclear transcriptional and developmental outputs through retrograde signaling networks [5].

In *Oryza sativa*, GPXs are best viewed as a five-member GPX family in canonical rice annotations rather than as a single generic antioxidant activity [6,7]. In plants, these enzymes belong to the cysteine-based phospholipid hydroperoxide GPX-type peroxidases and are functionally distinguished by subcellular targeting, developmental context, and redox partner preference [7,8]. Within this framework, GPX1 and GPX3 have been treated as organellar isoforms relevant to mitochondrial peroxide control in functional studies of japonica rice, supporting the idea that they contribute to local H_2_O_2_ buffering and redox signaling rather than merely to bulk detoxification [6,9]. Mechanistically, plant GPXs differ from the classical animal paradigm because they are thought to be reduced predominantly by thioredoxin-dependent systems, with possible contribution from glutaredoxin-linked pathways, instead of relying primarily on glutathione as the direct electron donor [7,8]. This isoform- and compartment-resolved view is important in rice because it provides the biological basis for hypothesizing that GPX1/GPX3 perturbation may be associated with changes in antioxidant metabolism, growth regulation, organellar-to-nuclear signaling, and DNA methylation-related plasticity [6,7,9].

Developmental transitions such as germination, growth establishment, and reproductive progression require ROS signals that are sufficiently robust to transmit information but sufficiently buffered to avoid chronic oxidative damage [1,10]. Recent work in *Arabidopsis thaliana*, for example, showed that exogenous ROS-generating treatments can reshape seed dormancy release, illustrating how developmental outputs can be shifted by changes in oxidative signaling thresholds [11]. More generally, organelle-derived signals are increasingly viewed as components of developmental control rather than as merely stress by-products [2,5]. On that basis, mitochondrial GPX insufficiency in rice may influence traits such as root growth, biomass accumulation, and flowering time through altered peroxide homeostasis, defective redox buffering, and disturbed retrograde communication [6,9].

An equally important, but less explored, dimension is the potential redox–epigenetic connection. In plants, methylome homeostasis emerges from the balance among maintenance methylation in CG, CHG, and CHH contexts, de novo methylation through RNA-directed DNA methylation (RdDM), and active demethylation by the ROS1/DME/DML DNA glycosylases acting through base-excision repair [12,13]. The literature also supports a homeostatic coupling between methylation and demethylation, such that demethylase expression—particularly ROS1—can itself depend on methylation at specific regulatory regions, effectively creating an epigenetic “thermostat” rather than a one-way silencing system [14]. Most importantly, recent environmental methylome work in Arabidopsis indicates that methylation plasticity often concentrates in non-CG space and TE-associated compartments rather than producing a uniform genome-wide gain or loss, with CHH changes particularly sensitive to RdDM/CMT2 balance [12,15]. This framework provides a basis for considering mitochondrial GPX knockdown as a potential source of DNA methylation-related changes, although such a connection remains a working hypothesis rather than a demonstrated mechanism. If mitochondrial redox imbalance impairs the thiol-redox network, oxidative stress may promote active remodeling of methylation states at stress-sensitive loci through DNA repair-linked demethylation pathways, whereas persistent redox disequilibrium may indirectly compromise methylation maintenance during replication, favoring passive dilution at selected genomic regions [12,13,14]. Either route could affect methylation-sensitive genomic compartments, especially in plants, where TE repression and epigenetic plasticity are deeply intertwined [12,15,16]. In monocots, recent work on reproductive phased small RNAs and DCL-pathway specialization shows that small-RNA systems linked to fertility and developmental robustness have undergone substantial diversification, including pathways centered on DCL4/DCL5-like functions and reproductive phasiRNAs [17,18]. While these pathways are not equivalent to canonical RdDM, they reinforce the broader conclusion that small-RNA-guided chromatin regulation is developmentally consequential in grasses.

Taken together, the recent evidence supports a working hypothesis in which mitochondrial GPX1/GPX3 silencing in rice should not be interpreted as a narrowly biochemical perturbation [6,9]. Rather, it may weaken the thiol-redox network [3,7], alter mitochondrial peroxide homeostasis, affect organellar signaling [4,5], and be associated with changes in DNA methylation-related measurements [12,13,14,15,16,17,19], with possible phenotypic consequences for growth and reproductive timing [6,9,10]. Guided by that framework, the present study evaluates whether silencing mitochondrial GPX1 and GPX3 in rice is associated with changes in antioxidant metabolism, plant developmental performance, total 5-methylcytosine content, and methylation-sensitive restriction profiles.

## 2. Results

### 2.1. GPX Silencing Impairs Early Establishment and Biomass Accumulation

**Figure 1:**
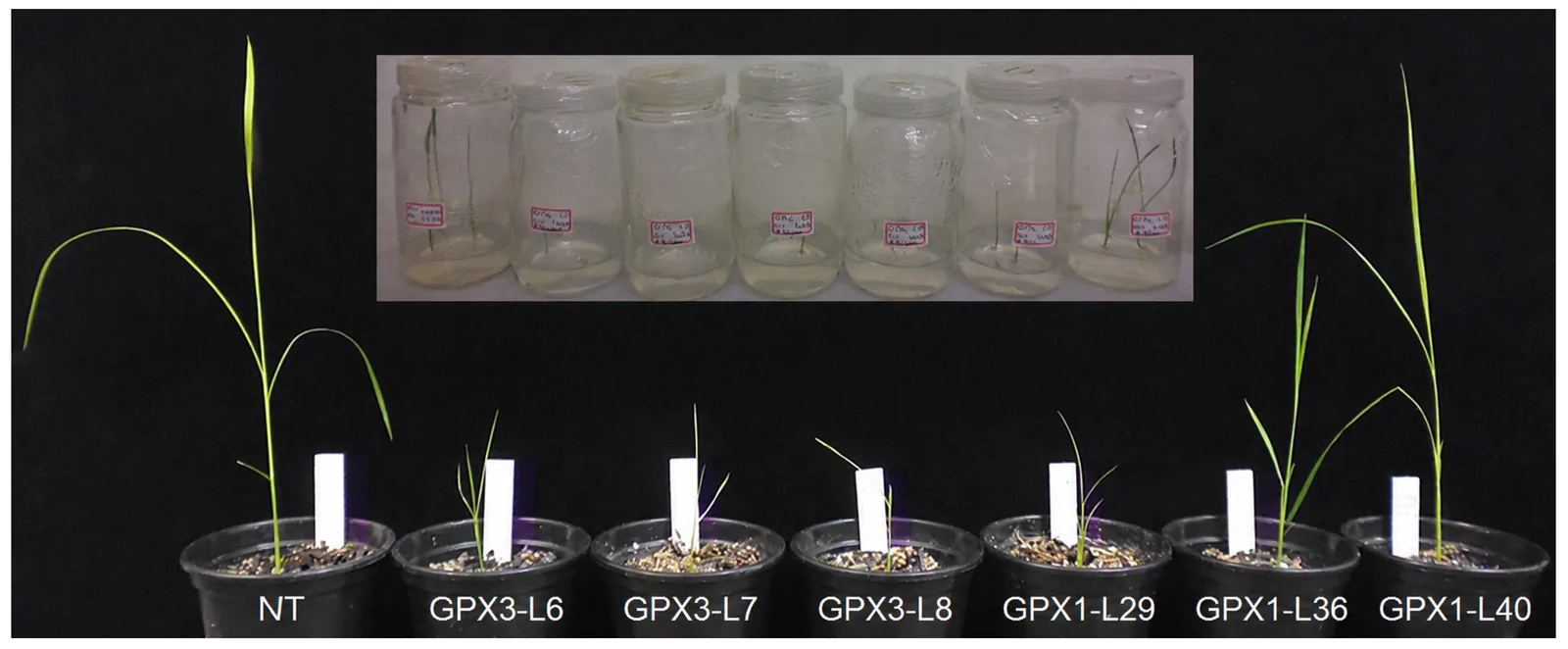
Early growth and acclimatization of NT plants and GPX-silenced rice lines during the transplanting stage.

During the transplanting and early acclimatization stage, clear phenotypic differences were observed among non-transformed Nipponbare plants and GPX-silenced rice lines (Figure 1). The GPX3-silenced lines showed the lowest early vigor, particularly GPX3-L6, GPX3-L7, and GPX3-L8, which exhibited reduced shoot development and weaker seedling establishment compared with NT plants (Figure 1). Because only one GPX3-L7 plant survived after early acclimatization, this line was considered only for qualitative observation at this stage and was not included in the subsequent morphophysiological or antioxidant enzyme statistical analyses. This response was especially evident during the transition from in vitro culture to substrate, indicating that early acclimatization represents a critical stage for GPX3-silenced seedlings (Figure 1). GPX1-silenced lines showed comparatively greater early shoot vigor during the transplanting stage, especially GPX1-L36 and GPX1-L40. However, this early advantage was not maintained throughout plant development, since GPX1-silenced lines later exhibited reduced biomass accumulation at harvest.

Analysis of variance showed significant genotype effects on flowering period, leaf biomass, root biomass, seed biomass, and total biomass, whereas tillering and stem biomass were not significantly affected (Table 1).

**Figure 2:**
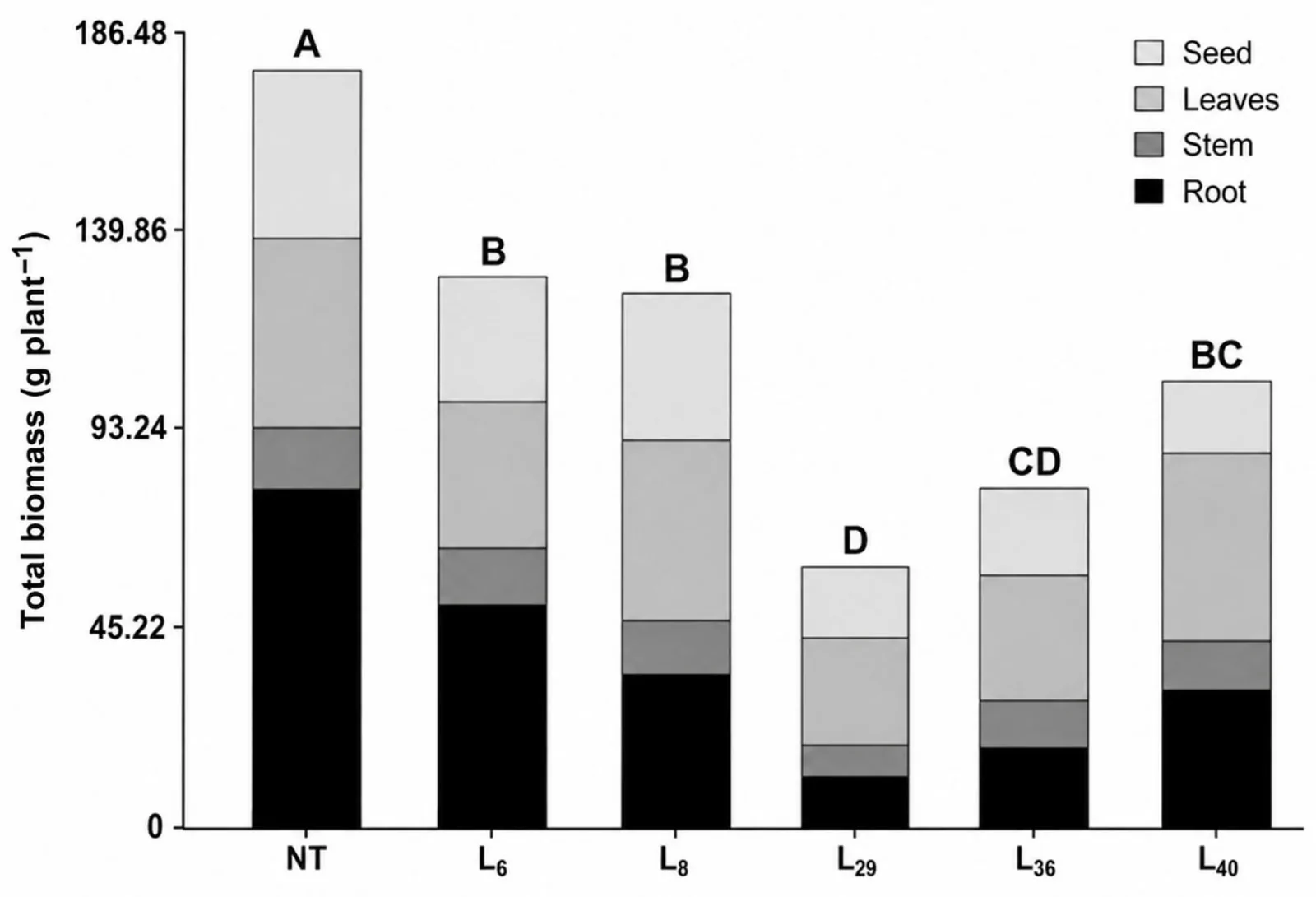
Biomass allocation at harvest in non-transformed and GPX-silenced rice lines. Stacked bars represent the contribution of root, stem, leaf, and seed biomass to total plant biomass in NT, GPX3-silenced lines (L6 and L8), and GPX1-silenced lines (L29, L36, and L40). Different letters above the bars indicate significant differences in total biomass among genotypes according to the mean comparison test at *p* < 0.05.

GPX gene silencing significantly reduced plant vigor, as reflected by lower biomass accumulation in all transformed rice lines compared with NT (Figure 2; Table 2). The reduction in total dry biomass was more pronounced in GPX1-silenced lines, particularly GPX1-L29 and GPX1-L36, which showed an average decrease of approximately 62.1% relative to NT plants. In contrast, GPX3-silenced lines exhibited a less severe reduction, with GPX3-L6 and GPX3-L8 showing an average decrease of approximately 29.2% in total biomass compared with NT plants (Figure 2). GPX1-L40 displayed an intermediate phenotype, reaching 99.3 g plant^−1^ of total dry biomass. Root biomass was the most strongly affected trait. Compared with NT plants, which produced 77.2 g plant^−1^ of root biomass, all GPX-silenced lines showed lower root biomass values (Table 2). The strongest reductions were observed in GPX1-L29 and GPX1-L36, with decreases of 86.1% and 78.5%, respectively (Table 2). GPX3-L6 and GPX3-L8 showed reductions of 35.5% and 57.6%, respectively, whereas GPX1-L40 exhibited a 64.6% reduction relative to NT plants (Table 2). Leaf biomass was less affected than root biomass and showed a line-dependent response. GPX1-L29 and GPX1-L36 had the most evident reductions in leaf biomass, decreasing by 43.4% and 35.0%, respectively, compared with NT plants (Table 2). In contrast, GPX3-L8 and GPX1-L40 maintained leaf biomass values similar to NT plants, with reductions of only 2.3% and 0.9%, respectively (Table 2). Seed biomass was also reduced in most GPX-silenced lines. Relative to NT plants, GPX1-L29, GPX1-L36, and GPX1-L40 showed decreases of 58.9%, 50.6%, and 58.7%, respectively, indicating that GPX1 silencing had a strong negative effect on reproductive biomass accumulation (Table 2). In GPX3-silenced lines, the effect was less severe, seed biomass decreased by 29.6% in GPX3-L6 and by 16.1% in GPX3-L8, with the latter remaining statistically comparable to NT plants.

In addition to biomass reduction, GPX silencing altered developmental timing. Flowering was delayed by 21.0 days in GPX3-L6, 30.7 days in GPX3-L8, and 28.7 days in GPX1-L29 relative to NT plants, corresponding to increases of 23.3%, 34.0%, and 31.8%, respectively. Overall, these results indicate that mitochondrial GPX knockdown was associated with impaired rice growth and altered developmental timing, with particularly strong effects on root biomass, reproductive biomass, and flowering time.

### 2.2. Reduced GPX-Associated Activity Is Linked to Selective Changes in the Antioxidant Defense System

Silencing of mitochondrial GPX genes altered plant developmental performance, particularly flowering time and biomass accumulation, in transgenic rice lines (Table 3). Analysis of variance showed that genotype had a significant effect (*p* < 0.05) on GR and GPX-associated activity, whereas CAT, APX, and SOD activities were not significantly affected by genotype (Table 4). According to Tukey’s test, NT plants showed the highest GPX-associated activity (254.1), while GPX1-L36 and GPX1-L40 showed significantly lower values, with activities of 149.2 and 148.1, respectively, corresponding to reductions of approximately 41.3% and 41.7% relative to NT plants (Table 4). GPX3-L6, GPX3-L8, and GPX1-L29 showed intermediate GPX-associated activity values and were not statistically separated from either NT plants or the most affected GPX1-silenced lines. A similar pattern was observed for GR activity: NT plants showed the highest value (1.5), whereas GPX3-L8, GPX1-L36, and GPX1-L40 showed significantly reduced GR activity, with decreases of approximately 40.0%, 33.3%, and 33.3%, respectively, compared with NT plants. Together, these results indicate that mitochondrial GPX gene silencing was associated with selective alteration of GPX-associated and glutathione-related antioxidant defense, rather than producing a generalized reduction across all antioxidant enzymes evaluated.

**Figure 3:**
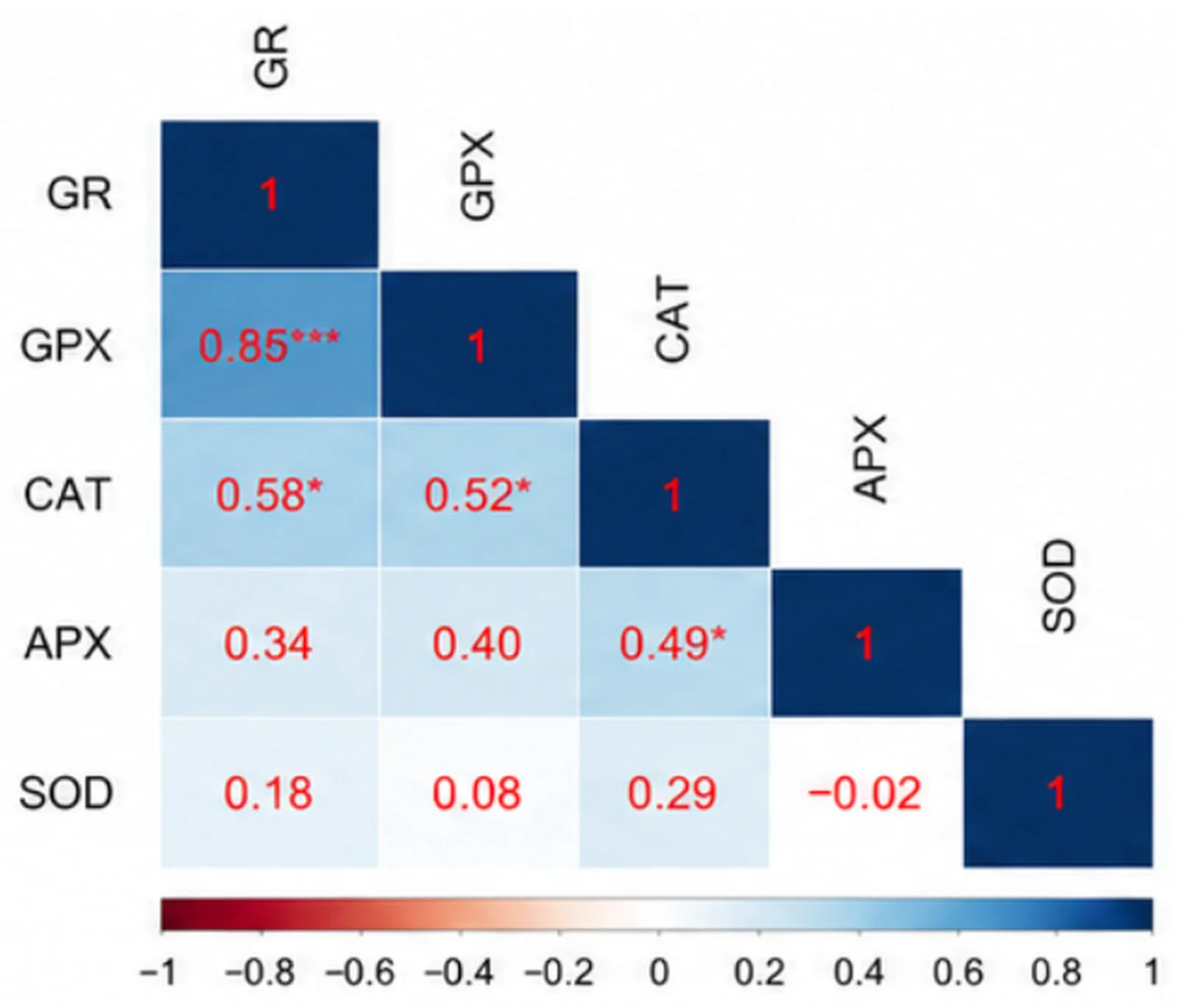
Pearson correlation matrix among antioxidant enzyme activities in non-transformed and GPX-silenced rice lines. The matrix shows pairwise correlations among glutathione reductase (GR), glutathione peroxidase-associated activity (GPX), catalase (CAT), ascorbate peroxidase (APX), and superoxide dismutase (SOD). * and *** indicate significant correlations at *p* < 0.05 and *p* < 0.001, respectively.

Correlation analysis revealed positive associations among several antioxidant enzyme activities (Figure 3). GPX-associated activity was strongly and positively correlated with GR activity (r = 0.85; *p* < 0.001), supporting a close association between GPX-associated peroxide-reducing capacity and glutathione-related redox responses under the assay conditions used. GPX-associated activity was also positively correlated with CAT activity (r = 0.52; *p* < 0.05). In addition, CAT activity was positively correlated with both GR activity (r = 0.58; *p* < 0.05) and APX activity (r = 0.49; *p* < 0.05). In contrast, SOD activity was not significantly associated with any of the other antioxidant enzymes evaluated. Taken together, these results indicate that GPX-silenced rice lines exhibit selective changes in GPX-associated and glutathione-related antioxidant defense, reflected by reduced GPX-associated and GR activities and their strong positive association.

### 2.3. GPX-Silenced Groups Exhibit Altered Total 5-Methylcytosine Content and Restriction-Sensitive Methylation Profiles

Reduced antioxidant capacity in GPX-silenced material was accompanied by differences in DNA methylation-related measurements. Because DNA methylation-related analyses were performed at the silencing-group level, NT, GPX1-silenced, and GPX3-silenced groups were compared. Analysis of variance showed that the silencing group had a significant effect on total 5-methylcytosine content and on methylation-dependent digestion by McrBC (*p* < 0.05), whereas digestion with the methylation-sensitive isoschizomers HpaII and MspI was not significantly affected (Table 5).

**Figure 4:**
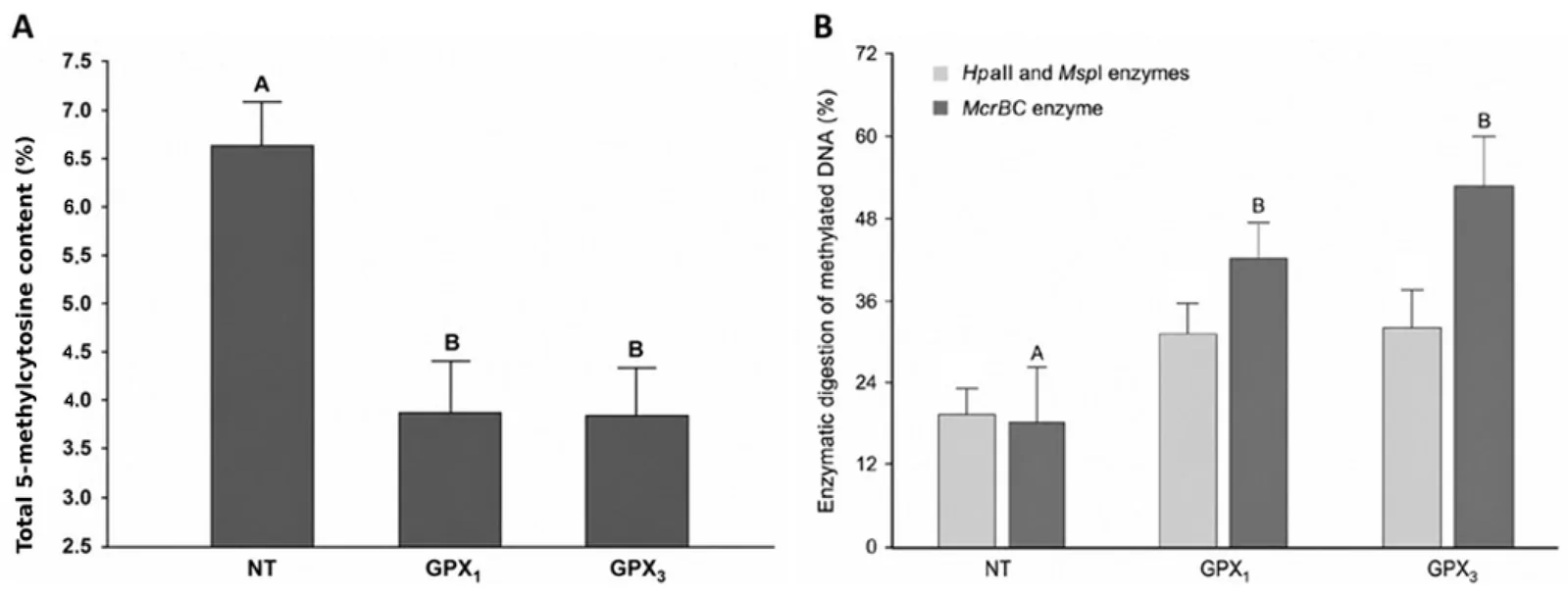
Total 5-methylcytosine content and methylation-sensitive restriction profiles in non-transformed and GPX-silenced rice groups. (**A**) Total 5-methylcytosine content in non-transformed plants (NT), GPX1-silenced, and GPX3-silenced groups. (**B**) Methylation-sensitive restriction digestion profiles obtained using HpaII/MspI and McrBC enzymes. Different letters indicate significant differences among groups within each analysis according to Tukey’s test at *p* < 0.05.

Total 5-methylcytosine content differed significantly among groups (*p* < 0.05), with NT plants showing the highest value, approximately 6.6% (Figure 4A). In contrast, GPX1- and GPX3-silenced groups showed markedly lower total 5-methylcytosine content, close to 3.9% and 3.8%, respectively (Figure 4A). This represented an estimated reduction of approximately 41.0% in the GPX1-silenced group and 42.0% in the GPX3-silenced group relative to NT plants. No significant difference was detected between GPX1- and GPX3-silenced groups, indicating that both knockdown groups showed comparable reductions in total 5-methylcytosine content under the assay conditions used. Restriction-sensitive digestion further indicated that methylation-related profiles differed depending on the enzymatic assay. Digestion with *HpaII* and *MspI*, which reflects methylation-sensitive variation at CCGG sites, did not differ significantly among NT, GPX1-, and GPX3-silenced groups (Figure 4B). In contrast, digestion with McrBC was significantly affected by the silencing group (*p* < 0.05). NT plants showed the lowest McrBC-based digestion value, approximately 18%, whereas GPX1- and GPX3-silenced groups showed higher values, close to 42% and 52%, respectively (Figure 4B). Relative to NT plants, this represented an increase of approximately 133% in the GPX1-silenced group and 189% in the GPX3-silenced group. Together, these results indicate that GPX-silenced groups were associated with reduced total 5-methylcytosine content, while simultaneously showing increased methylation-dependent restriction profiles detected by McrBC (Figure 4B). This suggests that reduced antioxidant capacity did not produce uniform genome-wide hypomethylation, but rather was associated with contrasting total 5-methylcytosine content and restriction-sensitive methylation profiles under the methods used.

## 3. Discussion

### 3.1. GPX Knockdown Is Associated with Impaired Growth and Altered Reproductive Timing

The results show that the reduction in antioxidant capacity associated with the silencing of mitochondrial GPX genes was accompanied by alterations in redox metabolism and by an impaired developmental phenotype, including reduced vigor during early establishment, lower biomass accumulation at the end of the growth cycle, and delayed transition to the reproductive phase [1,2]. Overall, this pattern is consistent with a scenario of chronic or insufficiently buffered oxidative stress, in which the plant must redirect resources from growth and reproduction toward cellular maintenance, repair, and homeostatic adjustment.

The loss of vigor observed in GPX-silenced lines represents one of the earliest phenotypic indications that disruption of redox balance compromised whole-plant development. During the transplanting and acclimatization stage, GPX3-silenced lines showed the poorest establishment, with reduced shoot development and lower capacity to adapt to the transition from in vitro conditions to substrate. In contrast, GPX1-silenced lines initially displayed greater vigor, although this response was not maintained until harvest (Figure 1, Supplementary Figure S1). At the whole-plant level, all transformed lines showed reduced total biomass compared with the control; however, this effect was particularly severe in the GPX1 lines. This response suggests that the mitochondrial isoforms GPX1 and GPX3 are not fully redundant but rather may contribute to different developmental windows or to tissues with distinct oxidative demands. The stronger biomass and root impairment observed in GPX1-silenced lines may reflect differences in expression level, tissue-specific function, developmental timing, insertional effects, or line-specific background variation. However, because GPX1 and GPX3 transcript abundance, protein abundance, and mitochondrial peroxide levels were not quantified in parallel in the present study, the greater apparent contribution of GPX1 to biomass maintenance remains hypothetical.

The disproportionate impairment of the root system is consistent with this interpretation. Because roots strongly depend on finely regulated ROS homeostasis to sustain cell division, elongation, and cell-wall remodeling, these results are biologically consistent with an imbalance between the signaling and toxic functions of H_2_O_2_ [3]. This molecule physiologically participates in pectin synthesis and demethylesterification in root apices; however, when antioxidant control is insufficient, excessive ROS may restrict meristematic activity, alter DNA replication, and reduce growth [4]. In this context, the decrease in root biomass likely acted as a physiological bottleneck for water and nutrient uptake, thereby amplifying the loss of vigor observed at the whole-plant level. Previous studies in rice have shown that GPX3 is essential for H_2_O_2_ homeostasis and for root and shoot development, and that chloroplastic and mitochondrial GPX genes play critical roles in crop development [5]. Moreover, oxidative stress can activate cell-cycle checkpoints and delay mitotic progression.

The reduction in seed biomass also indicates that the cost of redox imbalance was not restricted to vegetative growth but extended to biomass allocation toward reproductive organs. GPX1 lines reduced seed biomass by approximately 56%, whereas the effects in GPX3 lines were less pronounced. This pattern suggests that, in the lines with stronger oxidative impairment, GPX silencing may have altered source–sink relationships, metabolic stability during grain filling, and/or the continuity of post-transplant growth. In other words, reduced vigor does not appear to be a merely transient physiological response, but rather the manifestation of a sustained developmental penalty that ultimately affected reproductive performance. This link between ROS homeostasis and growth maintenance is also consistent with recent literature showing that ROS control is integrated with growth networks, stress tolerance, vascular development, and auxin signaling. In rice, even miR408-regulated circuits have been associated with plant architecture and auxin signaling, supporting the idea that subtle redox perturbations can lead to substantial changes in biomass accumulation and development [6].

The delayed flowering observed in several transformed lines adds a second dimension to the effect of oxidative stress. Oxidative stress not only reduced growth but also altered the pace of development. Delays of approximately 27 days compared with the NT control indicate a tendency of the transformed lines to prolong the vegetative phase. This response is consistent with the idea that, under oxidative pressure, cells prioritize maintenance and repair over differentiation and reproduction. Flowering also depends on the complex integration of energetic, hormonal, circadian, and environmental signals. Therefore, chronic disruption of mitochondrial H_2_O_2_ homeostasis may affect retrograde signaling pathways from organelles to the nucleus and, consequently, modify large-scale developmental programs. Recent studies have emphasized that organelle-dependent retrograde signaling participates in the coordination between stress and development, and that clock/environmental modules such as ELF3/ELF4 condition floral output in response to both external and internal signals [7]. Although florigenic genes and clock components were not quantified in the present study, the observed flowering delay is consistent with a possible developmental adjustment associated with oxidative stress.

### 3.2. GPX Silencing Selectively Disrupts Glutathione-Related Antioxidant Metabolism

On the other hand, our results indicate that silencing of mitochondrial GPX genes did not uniformly disrupt the entire antioxidant network but instead was associated with selective changes in GPX-associated and glutathione-related antioxidant defense. Indeed, the genotypic effect was significant only for GPX-associated activity and GR, whereas CAT, APX, and SOD did not show statistically significant differences among genotypes. Overall, this pattern is more consistent with a biochemical bottleneck in peroxide detoxification and in the regeneration of glutathione-based reducing power than with a generalized collapse of all antioxidant defenses. The strong positive correlation between GPX-associated activity and GR (*r* = 0.85; *p* < 0.001) further supports this interpretation. The coordinated decrease and strong positive association between GPX-associated and GR activities indicate linked variation within cellular thiol-redox metabolism; however, the assay does not establish a direct physiological electron-transfer pathway from GSH to GPX1 or GPX3. Recent literature has emphasized that the GSH/Grx system occupies a central position in cellular redox homeostasis, reversible protein S-glutathionylation, and the fine regulation of redox signaling [8]. Therefore, a concomitant decrease in GPX-associated activity and GR should not be interpreted merely as an enzymatic change, but rather as evidence of altered GPX-associated and glutathione-related redox responses under the assay conditions used. Within this framework, the simultaneous decrease observed in our transformed lines suggests that GPX silencing shifted the system toward a less flexible state, reducing its capacity to buffer oxidative pulses and sustain glutathione-related signaling responses.

The absence of significant differences in CAT, APX, and SOD is also informative. Far from weakening the effect of gene silencing, it suggests that the primary alteration did not occur across the entire ROS-scavenging network, but rather within a specific antioxidant module. The positive correlations of CAT with GR, GPX-associated activity, and APX are compatible with a certain degree of functional connectivity or second-order compensation. However, the fact that CAT did not vary significantly among genotypes indicates that this connectivity was not sufficient to compensate for the primary defect. In addition, SOD was not significantly associated with any of the other enzymes, suggesting that the limiting step was not superoxide dismutation, but rather downstream processing of H_2_O_2_ and/or hydroperoxides through GPX-associated and glutathione-related antioxidant defense. This interpretation is consistent with the current view that ROS responses in plants are organized around specific redox nodes and localized modules [9], rather than through parallel and equivalent regulation of all antioxidant components.

The specificity of this effect is even more relevant because the silenced genes correspond to mitochondrial isoforms. In this context, the reduction in GPX-associated activity and GR may be interpreted as a loss of capacity to locally control the redox state of an organelle that not only produces ROS, but also participates in the integration of metabolism, growth, and signaling toward the nucleus. Although mitochondrial ROS, GSH/GSSG ratios, and retrograde signaling markers were not directly quantified in this study, the association between biochemical impairment of the GPX/GR system and the phenotypes of reduced biomass and delayed flowering observed in the same lines is consistent with the idea that organellar redox deregulation may be linked to developmental changes at the whole-plant scale. Recent plant studies have convincingly shown, at least for plastid retrograde signaling, that communication from organelles can condition developmental progression and modulate transitions between physiological states [8,10]. In our lines, mitochondrial GPX silencing may have acted as a restricting factor of the growth program, even though the specific molecular mechanism remains to be resolved.

### 3.3. GPX Knockdown Is Associated with Altered Total 5-Methylcytosine Content and Restriction-Sensitive Methylation Profiles

Silencing of mitochondrial GPX1 and GPX3 was associated with significant, but contrasting, changes in DNA methylation-related measurements. Total 5-methylcytosine content decreased by approximately 41% in the knockdown groups, whereas susceptibility to McrBC digestion increased and HpaII/MspI-sensitive profiles remained statistically unchanged. These findings indicate that the methylation response was not equally detected by all analytical approaches. However, because the assays differ in sequence recognition and methylation sensitivity, the current data cannot determine whether the contrasting profiles reflect locus-specific hypermethylation, changes in cytosine context, altered spacing of methylated sites, or other differences in genomic methylation organization.

Within this framework, at least two classes of mechanisms may be considered as possible explanations for the observed results. The first involves active DNA demethylation, which in plants is primarily mediated by the DNA glycosylases ROS1, DME, DML2, and DML3 through the base-excision repair pathway. The second involves passive demethylation, defined as the progressive loss of methylation when epigenetic marks are not efficiently restored following DNA replication. Both mechanisms are biologically plausible in the transformed lines. Persistent oxidative stress, together with impairment of the GPX-associated and glutathione-related antioxidant response, could potentially influence active demethylation or methylation maintenance pathways associated with DNA repair and genome surveillance [11]. At the same time, oxidative imbalance could be associated with reduced efficiency of methylation maintenance over successive cell divisions, resulting in dilution of methylation marks in some genomic regions [12]. Plant studies further indicate that ROS1 participates in a homeostatic circuit through which DNA methylation and demethylation machineries mutually regulate one another. A sustained redox disturbance could therefore disrupt this epigenetic “rheostat” without affecting all cytosine contexts equally. Because the present data were obtained using total 5-methylcytosine quantification and restriction enzyme-based assays, it is not possible to determine conclusively how much of the observed reduction in total 5-methylcytosine content resulted from active demethylation and how much reflected passive loss caused by incomplete maintenance. Nevertheless, the observed reduction in total 5-methylcytosine content is compatible with either active demethylation, incomplete maintenance during replication, or a combination of both processes.

The magnitude of the decrease in total 5-methylcytosine content should be interpreted cautiously. A reduction from approximately 6.6% to 3.8% indicates a substantial difference in total 5-methylcytosine signal under the assay conditions used. However, because the colorimetric assay does not resolve cytosine context, locus identity, or genomic compartment, this decrease should not be interpreted as uniform genome-wide hypomethylation. Instead, it should be considered an initial, low-resolution indication of altered total 5-methylcytosine content that requires validation by whole-genome bisulfite sequencing, enzymatic methyl sequencing, or other locus-resolved approaches.

The contrasting results obtained from total 5-methylcytosine quantification and methylation-sensitive restriction assays suggest that GPX knockdown did not affect all methylated sites uniformly. Because *McrBC*, *HpaII*, and *MspI* differ in sequence recognition and methylation sensitivity, these profiles may reflect differences in the abundance, spacing, or sequence context of methylated cytosines. Plant methylation responses are frequently enriched in non-CG and transposable-element-associated regions, particularly through RNA-directed DNA methylation pathways [13,14,20]. However, the present analyses do not identify the genomic compartments responsible for the observed differences. Therefore, the possible involvement of repetitive or transposable-element-associated regions should be regarded as a hypothesis requiring locus-resolved methylome analysis.

The observed DNA methylation-related differences also raise the possibility that transposable-element-associated regions may be affected, because these genomic compartments are highly responsive to non-CG methylation and RdDM-mediated regulation [13,14,15]. Nevertheless, TE transcription, small-RNA populations, insertion polymorphisms, and transmission to the progeny were not evaluated in this study. Consequently, no conclusion can be drawn regarding TE derepression, mobilization, or heritable genetic variation. Testing this hypothesis will require context-resolved methylome profiling together with TE-expression, small-RNA, and insertion-site analyses.

These findings support an association between reduced mitochondrial antioxidant capacity and altered DNA methylation-related measurements. The concurrence of reduced GPX- and GR-associated activities, lower vigor, delayed flowering, and changes in total 5-methylcytosine content and restriction-sensitive methylation measurements suggests a potential association between cellular redox status and epigenetic regulation [21]. In this context, DNA methylation may represent an interface linking metabolism, stress responses, and plant development. However, the present data do not establish whether the methylation changes occurred in genes, regulatory regions, repetitive DNA, or transposable elements, nor whether they directly contributed to the observed developmental phenotypes.

### 3.4. Open Questions and Limitations

The principal limitation of the present study is its genomic resolution. Total 5-methylcytosine quantification and restriction enzyme-based assays cannot assign the observed changes to CG, CHG, or CHH contexts, nor can they establish whether the affected loci correspond to genes, promoters, transposable elements, TE boundaries, or pericentromeric heterochromatin. These approaches also cannot determine whether active, passive, or combined demethylation mechanisms predominated, or whether transposable elements became transcriptionally active and produced heritable consequences [22]. The most decisive next steps would therefore include whole-genome bisulfite sequencing or enzymatic methyl sequencing to resolve cytosine-context-specific changes. The absence of these molecular layers does not invalidate the central interpretation, but it requires the proposed mechanisms to be presented as biologically plausible rather than experimentally demonstrated.

A second limitation is that antioxidant enzyme activities were measured at a single developmental stage, 60 days after transplantation. Because redox metabolism is highly dynamic and varies with tissue, developmental stage, and environmental conditions, the enzyme activities reported here represent a snapshot of redox status at that sampling time. Therefore, the observed differences should not be interpreted as a complete temporal description of the antioxidant response. Future studies should include multiple developmental stages and tissues to determine whether GPX-associated and glutathione-related responses are transient, sustained, or developmentally regulated.

A further limitation is that the transformed lines originated from tissue culture-derived material. Somaclonal variation, including epigenetic variation induced during in vitro culture and regeneration, cannot be completely excluded. This is particularly relevant for the DNA methylation-related measurements. Although multiple independently transformed lines were used, the absence of regenerated empty-vector controls prevents us from excluding tissue culture- or transformation-associated epigenetic differences that may have pre-existed before the present evaluation.

In addition, complementation or rescue experiments were not performed. Therefore, the observed phenotypes should be interpreted as associated with GPX knockdown in the available transgenic material rather than as definitive proof of direct causality. Similarly, direct H_2_O_2_ measurements, mitochondrial ROS quantification, GSH/GSSG ratios, and expression analyses of methylation and demethylation regulators such as ROS1, DME/DML, MET1, CMT3, and DRM2 were not included. These measurements represent critical next steps to test whether the methylation-related differences observed here are mechanistically connected to redox imbalance.

Finally, correlations between antioxidant enzyme activities, morphophysiological traits, and DNA methylation-related measurements should be interpreted cautiously because the methylation dataset was generated at the silencing-group level with limited replication and did not fully match the line-level structure of the morphophysiological and antioxidant datasets. Therefore, the current study supports associations among redox status, plant performance, and DNA methylation-related measurements, but does not establish causal relationships among these layers.

## 4. Materials and Methods

### 4.1. Plant Material and Experimental Design

The study was conducted at the Cytogenomics and Epigenetics Laboratory (CYNGELA), Department of Genetics, Luiz de Queiroz College of Agriculture, University of São Paulo (ESALQ/USP), Piracicaba, São Paulo, Brazil. Rice (*Oryza sativa* L. ssp. japonica) plants of the cultivar Nipponbare were used as the non-transformed control (NT). The transgenic rice lines carrying knockdown constructs targeting the mitochondrial glutathione peroxidase genes GPX1 and GPX3 were kindly provided by Prof. Dr. Márcia Margis-Pinheiro, Federal University of Rio Grande do Sul, Porto Alegre, Rio Grande do Sul, Brazil. The construction of the silencing vectors, rice transformation, hygromycin selection, and molecular validation of reduced GPX1 and GPX3 expression were described previously by Passaia et al. [3,4] and Passaia and Margis-Pinheiro [4]. The present study did not generate the transformation constructs or the original transgenic events; instead, it used these previously established lines to evaluate morphophysiological performance, antioxidant enzyme activities, and DNA methylation profiles.

Six independently transformed rice lines were initially established: GPX1-L29, GPX1-L36, and GPX1-L40, carrying silencing constructs targeting GPX1; and GPX3-L6, GPX3-L7, and GPX3-L8, carrying silencing constructs targeting GPX3. The GPX3-L7 line showed severe loss of vigor during early post-transplant acclimatization, and only one plant survived. Consequently, this line was excluded from the subsequent morphophysiological and antioxidant enzyme analyses; however, the surviving plant provided sufficient leaf material for DNA extraction and was retained for the DNA methylation analyses. Therefore, five transformed lines were retained for the morphophysiological and antioxidant enzyme analyses, whereas all six independent transformation events were represented in the DNA methylation analysis. Non-transformed Nipponbare plants were included as the reference genotype.

The experimental strategy involved two related but distinct analytical approaches. For morphophysiological traits and antioxidant enzyme activities, the experiment was arranged as a randomized complete block design under greenhouse conditions. Three blocks were established, and each block contained one experimental unit of each genotype. Each experimental unit consisted of one individual plant grown in a separate pot. Thus, five GPX-silenced lines and the non-transformed Nipponbare control were evaluated with three biological replicates per genotype, corresponding to one plant per genotype in each block. Plants were randomly assigned to positions within each block to minimize positional effects associated with greenhouse microenvironmental variation. All plants were maintained under the same substrate, irrigation, fertilization, acclimatization, and greenhouse management conditions.

For DNA methylation analyses, the experimental unit was defined differently because the objective was to compare the methylation response associated with the target mitochondrial GPX isoform rather than the performance of each individual line. Therefore, independently transformed lines were treated as independent biological replicates within each silencing group. The GPX1-silenced group included GPX1-L29, GPX1-L36, and GPX1-L40, whereas the GPX3-silenced group included GPX3-L6, GPX3-L7, and GPX3-L8. Three randomly selected non-transformed Nipponbare plants were used as biological replicates for the NT group.

### 4.2. Seed Disinfection, Germination, and Selection of Transformed Plants

Rice seeds were manually dehulled and surface-disinfected by immersion in 70% ethanol for 30 s, followed by three washes with sterile distilled water. Seeds were subsequently immersed in a 3:1 mixture of distilled water and commercial sodium hypochlorite solution containing 2.5% available chlorine. The suspension was gently stirred using a magnetic stirrer, after which the seeds were washed three times with sterile distilled water under aseptic conditions.

Disinfected seeds were transferred to glass culture vessels containing approximately 50 mL of Murashige and Skoog basal medium without plant growth regulators. The medium contained 30 g L^−1^ sucrose, and its pH was adjusted to 5.8 before autoclaving at 120 °C and 20 psi for 15 min.

For selection of transformed plants, hygromycin was filter-sterilized through a 0.22 µm membrane and added to the cooled culture medium. Seeds of the non-transformed Nipponbare control were germinated on the same medium without hygromycin. Seedlings showing severe chlorosis, poor growth, or loss of pigmentation under hygromycin selection were discarded.

### 4.3. Seedling Acclimatization and Greenhouse Growth Conditions

Seedlings were transferred from the culture medium when they reached approximately 6 cm in height. Their roots were gently washed with water to remove residual medium, and the seedlings were transplanted into containers holding approximately 500 g of a 1:1 soil-to-sand mixture. Each plant received approximately 20 mL of half-strength Murashige and Skoog nutrient solution immediately after transplantation.

The containers were covered with transparent plastic bags to maintain high relative humidity during acclimatization. The bags were removed after 2 days. Following initial acclimatization, vigorous plants were transplanted into pots containing approximately 1 kg of a 1:1:1:1 mixture of soil, sand, vermiculite, and humus. Plants remained in the acclimatization room for an additional 4 days and were subsequently transferred to greenhouse conditions.

All genotypes were cultivated under the same environmental and management conditions. A balanced N–P–K fertilizer formulation of 20–20–20 was supplied monthly to minimize nutritional stress.

### 4.4. Morphophysiological Characterization

Morphophysiological traits were evaluated individually for each plant. Days to flowering were recorded as the number of days between sowing and the appearance of reproductive structures. Tillering was determined by counting the total number of tillers per plant at flowering.

At physiological maturity, plants were harvested and separated into leaves, stems, roots, and seeds. Roots were carefully recovered from the substrate and washed to remove adhering particles. Plant fractions were placed separately in labeled paper bags and oven-dried at 60 °C until a stable dry mass was obtained. Leaf, stem, root, and seed dry biomass were recorded in grams per plant. Total biomass was calculated as the sum of all dry plant fractions. Relative chlorophyll content was determined at harvest using a portable SPAD-502 Plus chlorophyll meter (Konica Minolta, Tokyo, Japan).

### 4.5. Sampling and Preparation of Plant Extracts for Antioxidant Enzyme Assays

Fully expanded, nonsenescent leaves from the middle third of each plant were collected 60 days after transplantation. Leaves of comparable position and physiological condition were selected to minimize variation associated with leaf age, developmental status, and senescence. Samples were immediately frozen in liquid nitrogen and stored under frozen conditions until biochemical analysis.

Leaf tissue was homogenized under chilled conditions, and the resulting protein extracts were used to quantify the activities of glutathione peroxidase-associated activity (GPX), glutathione reductase (GR), ascorbate peroxidase (APX), catalase (CAT), and superoxide dismutase (SOD). All spectrophotometric assays were conducted using at least three technical measurements, and enzyme activities were normalized to the soluble protein concentration of each extract.

### 4.6. Soluble Protein Quantification

Soluble protein concentration was determined using the Bradford method with a commercial Bio-Rad protein assay reagent. Bovine serum albumin was used to construct a calibration curve ranging from 0.1 to 1.0 mg mL^−1^.

For each determination, 20 µL of the standard or plant extract was mixed with 1 mL of Bradford reagent. Samples were incubated for 20 min, and absorbance was measured at 595 nm. Protein concentrations were calculated from the linear regression equation obtained from the bovine serum albumin calibration curve.

### 4.7. Glutathione Peroxidase-Associated Activity

GPX-associated activity was determined using hydrogen peroxide as the substrate in a coupled reaction containing reduced glutathione, glutathione reductase, and NADPH. Enzyme activity was estimated by monitoring NADPH oxidation as a decrease in absorbance at 340 nm.

The assay mixture contained potassium phosphate buffer, 3 mM EDTA, 10 mM reduced glutathione, 1 mM sodium azide, plant protein extract, NADPH, and hydrogen peroxide. The reaction mixture was preincubated at 37 °C for 5 min. The reaction was performed in a final volume of 1 mL containing 775 µL of the basal reaction mixture, 25 µL of protein extract, 100 µL of NADPH solution, and 100 µL of hydrogen peroxide solution.

The change in absorbance was recorded for 1 min. GPX-associated activity was calculated using an NADPH molar extinction coefficient of 6.22 mM^−1^ cm^−1^ and expressed as nmol NADPH oxidized min^−1^ mg^−1^ protein.

### 4.8. Glutathione Reductase Activity

GR activity was determined according to the reduction of oxidized glutathione coupled to the formation of 5-thio-2-nitrobenzoic acid. The reaction medium contained 100 mM potassium phosphate buffer, pH 7.5, 1 mM 5,5′-dithiobis-(2-nitrobenzoic acid), 1 mM oxidized glutathione, and 0.1 mM NADPH.

The reagents were maintained on ice and protected from light before use. Reaction cuvettes were equilibrated at 30 °C. The reaction was initiated by adding 50 µL of plant protein extract, and absorbance was monitored at 412 nm for 1 min. A corresponding blank was prepared by replacing the protein extract with potassium phosphate buffer. GR activity was normalized to protein concentration and expressed as µmol min^−1^ mg^−1^ protein.

### 4.9. Ascorbate Peroxidase Activity

APX activity was quantified by monitoring the oxidation of ascorbate at 290 nm. The reaction mixture contained 80 mM potassium phosphate buffer, pH 7.0, 5 mM ascorbate, 1 mM EDTA, 1 mM hydrogen peroxide, and plant protein extract.

The reaction components were maintained at 30 °C and protected from light. The reaction was initiated by adding 50 µL of plant extract to the assay mixture. Absorbance was recorded for 1 min in quartz cuvettes. A reaction blank was prepared by replacing the plant extract with potassium phosphate buffer. APX activity was calculated using an extinction coefficient of 2.8 mM^−1^ cm^−1^ and expressed as nmol ascorbate oxidized min^−1^ mg^−1^ protein.

### 4.10. Catalase Activity

CAT activity was determined by monitoring the decomposition of hydrogen peroxide at 240 nm. The assay medium consisted of 100 mM potassium phosphate buffer, pH 7.5, supplemented with hydrogen peroxide.

The reaction was initiated by adding 25 µL of plant protein extract to 1 mL of reaction medium. Absorbance was recorded at 1 s intervals for 1 min at 25 °C using quartz cuvettes. All reagents and reactions were protected from direct light. A blank was prepared by replacing the plant extract with phosphate buffer.

CAT activity was calculated using the molar extinction coefficient of hydrogen peroxide, 39.4 M^−1^ cm^−1^, and expressed as µmol H_2_O_2_ decomposed min^−1^ mg^−1^ protein.

### 4.11. Superoxide Dismutase Activity

SOD activity was determined from its ability to inhibit the photochemical reduction of nitroblue tetrazolium. The 3 mL reaction mixture contained 50 mM sodium phosphate buffer, pH 7.8, EDTA, methionine, nitroblue tetrazolium, riboflavin, and 50 µL of plant protein extract.

The reaction mixtures were exposed to light for 5 min, and absorbance was subsequently measured at 560 nm. A non-illuminated reaction was used as the blank. One unit of SOD activity was defined as the amount of enzyme required to inhibit nitroblue tetrazolium photoreduction by 50%. Activity was expressed as SOD units mg^−1^ protein.

### 4.12. Genomic DNA Extraction

Genomic DNA was extracted from 500 to 600 mg of leaf tissue using a cetyltrimethylammonium bromide protocol. Leaf samples were ground to a fine powder in liquid nitrogen and transferred to preheated extraction buffer containing 1% β-mercaptoethanol. Samples were incubated at 65 °C for 20–30 min.

An equal volume of chloroform:isoamyl alcohol solution (24:1, *v*/*v*) was added, and samples were gently mixed for 5 min before centrifugation at 13,000 rpm for 15 min. The aqueous phase was transferred to a new tube and treated with RNase at 37 °C. DNA was precipitated with isopropanol, recovered by centrifugation, washed, and resuspended in TE buffer.

A second purification was performed using 7.5 M ammonium acetate and absolute ethanol. DNA pellets were washed with 70% ethanol, air-dried, and resuspended in TE buffer. DNA integrity and concentration were evaluated by electrophoresis on 1% agarose gels using a molecular-weight standard.

### 4.13. Total 5-Methylcytosine Quantification

For DNA methylation analyses, plants were randomly selected from the GPX1-knockdown, GPX3-knockdown, and NT groups. The independently transformed lines were considered independent experimental replicates within their corresponding target-gene group. Three biological replicates were included per group. Each GPX1 and GPX3 line (GPX1: L29, L36, L40 and GPX3: L6, L7, L8) contributed equally to the analysis. Although only one GPX3-L7 plant survived the early acclimatization stage, this plant provided sufficient leaf tissue for DNA extraction and was included as the biological replicate representing the L7 transformation event. Three randomly selected NT plants were included as biological replicates of the NT control.

Total genomic 5-methylcytosine content was quantified using a commercial colorimetric enzyme-linked immunosorbent assay according to the manufacturer’s instructions. Equal amounts of purified genomic DNA were analyzed for each biological replicate. Absorbance values were converted to percentages of 5-methylcytosine using the positive and negative controls supplied with the kit.

This assay provides an estimate of total genomic 5-methylcytosine content under the assay conditions used. It does not resolve methylation at specific loci, distinguish cytosine sequence contexts (CG, CHG, or CHH), or identify the genomic compartments contributing to the signal. Therefore, the results were interpreted as total 5-methylcytosine measurements rather than as genome-wide, context-resolved methylome profiles.

### 4.14. Methylation-Sensitive Restriction Analysis with HpaII and MspI

Methylation-sensitive restriction analysis was conducted using the isoschizomers HpaII and MspI, which recognize the same 5′-CCGG-3′ sequence but differ in their sensitivity to methylation.

For each genotype, 1 µg of genomic DNA was digested separately with 1 U of HpaII or MspI (New England Biolabs, Ipswich, MA, USA). Reactions were incubated at 37 °C for 16 h. The enzymes were subsequently heat-inactivated at 95 °C for 10 min, and digested DNA was stored at −20 °C until analysis. Restriction profiles were evaluated by electrophoresis on 1% agarose gels.

The HpaII/MspI profiles were interpreted comparatively based on the relative digestion pattern observed among NT, GPX1-silenced, and GPX3-silenced groups. Because HpaII and MspI recognize the same CCGG sequence but differ in methylation sensitivity, differences in banding or digestion intensity were considered indicative of methylation-sensitive variation at CCGG sites. However, this assay does not identify the affected loci, does not resolve cytosine sequence context outside the recognized CCGG sites, and should therefore be interpreted as a restriction-sensitive profile rather than as a genome-wide methylation measurement.

### 4.15. McrBC Digestion Assay

Methylation-dependent DNA digestion was performed using McrBC, which preferentially cleaves DNA containing methylated cytosines. For each reaction, 1 µg of genomic DNA was combined with 0.5 µL of McrBC enzyme, 4 µL of the corresponding reaction buffer, 4 µL of bovine serum albumin solution, and 0.5 µL of GTP.

Samples were incubated at 37 °C for 16 h, followed by enzyme inactivation at 95 °C for 10 min. Digested samples were stored at −20 °C and subsequently separated by electrophoresis on 1% agarose gels. Gel images were analyzed using ImageJ 1.54p (National Institutes of Health, Bethesda, MD, USA) to estimate the relative proportion of digested DNA.

For McrBC, higher relative digestion was interpreted as greater susceptibility of methylated DNA to methylation-dependent cleavage under the assay conditions used, whereas lower digestion indicated lower relative susceptibility. Because McrBC cleavage depends on the presence and spacing of methylated cytosines, the resulting profiles were interpreted as methylation-dependent restriction patterns rather than as direct measures of total methylation abundance or locus-specific methylation. Therefore, contrasting results between total 5-methylcytosine quantification, HpaII/MspI digestion, and McrBC digestion were interpreted as evidence of nonuniform methylation-related differences detectable by the specific assays used.

### 4.16. Statistical Analysis

Morphophysiological traits and antioxidant enzyme activities were analyzed according to a randomized complete block model:

Y_ij_ = μ + G_i_ + B_j_ + ε_ij_

where Y_ij_ is the observed value of the i-th genotype in the j-th block; μ is the overall mean; G_i_ is the fixed effect of the i-th genotype; B_j_ is the effect of the j-th block; and ε_ij_ is the random experimental error, assumed to be independently and normally distributed with a mean of zero and constant variance [ε_ij_~N(0, σ^2^)]. For these analyses, each GPX-silenced line and the NT control were treated as individual genotypes, and each individual plant represented one experimental unit.

For DNA methylation analyses, a separate one-way analysis of variance was performed using the silencing group as the main factor. In this case, the groups were NT, GPX1-silenced, and GPX3-silenced. Independently transformed lines were considered independent biological replicates within each GPX-silenced group, whereas randomly selected NT plants represented the biological replicates of the control group. Data were subjected to analysis of variance after verification of model assumptions. When a significant genotype or group effect was detected, means were compared using Tukey’s honestly significant difference test at *p* < 0.05.

Pearson correlation coefficients were calculated among GPX, GR, APX, CAT, and SOD activities. Statistical analyses were performed using R software R4.6.0 [23].

## 5. Conclusions

Mitochondrial GPX1 and GPX3 knockdown was associated with impaired rice growth, altered reproductive timing, and selective changes in GPX-associated and glutathione-related antioxidant defense. GPX-silenced lines exhibited reduced early vigor and lower biomass accumulation, particularly in roots and seeds, and delayed flowering. The biochemical response was selective, as genotype significantly affected GPX-associated activity and GR without significantly altering CAT, APX, or SOD activities.

GPX-silenced groups also showed reduced total 5-methylcytosine content and contrasting restriction-sensitive methylation profiles, characterized by increased McrBC digestion and unchanged HpaII/MspI profiles. These results should not be interpreted as evidence of uniform genome-wide hypomethylation, but rather as preliminary evidence of DNA methylation-related differences detected by low-resolution and restriction-based approaches.

Overall, these findings support an association among mitochondrial GPX knockdown, redox metabolism, developmental performance, and DNA methylation-related measurements in rice. However, the epigenetic component should be considered exploratory because the present analyses do not resolve cytosine context, genomic loci, or causal mechanisms. Context-resolved methylome profiling, direct redox measurements, and expression analyses of methylation and demethylation regulators will be required to test the proposed redox–epigenetic relationship.

## Figures and Tables

**Table 1:** Mean squares for flowering time, tillering, and biomass-related traits in rice lines.

Source of Variation	df	Flowering Period	Tillering	Leaf Biomass	Stem Biomass	Root Biomass	Seed Biomass	Total Biomass
Model	7	704.11	41.89	267.77	23.54	1559.65	180.58	379.89
Genotypes	5	486.90 *	53.82 ns	210.49 **	32.77 ns	1791.27 **	203.73 **	377.56 *
Block	2	1247.16 **	12.06 ns	410.98 **	0.45 ns	980.59 *	122.72 **	385.70 *
Error	10	94.17	27.39	33.85	10.55	220.93	13.11	72.63
CV (%)	—	9.11	30.39	15.98	27.17	41.62	23.62	17.62

* Significant at *p* < 0.05; ** significant at *p* < 0.01; ns = not significant.

**Table 2:** Morphophysiological performance of non-transformed and GPX-silenced rice lines.

Genotype/Line	Root Biomass	Leaf Biomass	Seed Biomass	Days to Flowering
NT	77.2 a	44.0 a	40.9 a	90.3 c
GPX3-L6	49.8 b	34.4 ab	28.8 bc	111.3 ab
GPX3-L8	32.7 bc	43.0 a	34.3 ab	121.0 a
GPX1-L29	10.7 c	24.9 b	16.8 d	119.0 a
GPX1-L36	16.6 c	28.6 b	20.2 cd	94.3 bc
GPX1-L40	27.3 bc	43.6 a	16.9 d	103.0 bc

Values represent means from three replicates. Different lowercase letters within the same column indicate significant differences among genotypes according to Tukey’s test at *p* < 0.05.

**Table 3:** Mean squares of antioxidant enzyme activities in six rice genotypes.

Source of Variation	df	CAT	GR	APX	SOD	GPX
Genotype	5	23,557.26 ns	0.15 *	1871.80 ns	73.37 ns	5069.09 *
Block	2	8922.07 ns	0.17 *	424.19 ns	72.64 ns	6698.92 *
Error	10	9948.02	0.03	889.27	52.22	1286.76
CV (%)	—	30.17	15.23	46.16	52.67	20.53

* Significant *p* < 0.05; ns not significant differences.

**Table 4:** Influence of knockdown GPX genes on antioxidant enzyme activity of six rice genotypes.

Antioxidant Enzyme	GPX	GR
NT	254.1 a	1.5 a
GPX3-L6	152.4 ab	1.1 ab
GPX3-L8	160.5 ab	0.9 b
GPX1-L29	184.1 ab	1.2 ab
GPX1-L36	149.2 b	1.0 b
GPX1-L40	148.1 b	1.0 b

Values represent means from three replicates. Different lowercase letters within the same column indicate significant differences among genotypes according to Tukey’s test at *p* < 0.05.

**Table 5:** Analysis of variance for total 5-methylcytosine content and restriction-sensitive methylation profiles in NT, GPX1-silenced, and GPX3-silenced groups.

Source of Variation	df	Total 5-Methylcytosine Content (%)	DNA Digestion by *McrBC*	DNA Digestion by *HpaII* and *MspI* (%)
Silencing group	2	5.30 *	0.09 *	0.01 ns
Error	6	0.73	0.01	0.001
CV (%)	—	18.7	29.3	19.4

* Significant *p* < 0.05; ns not significant differences.

## Data Availability

The original contributions presented in this study are included in the article. Further inquiries can be directed to the corresponding author.

## Supplementary Materials

The following supporting information can be downloaded at: https://www.mdpi.com/article/10.3390/epigenomes10030049/s1, Figure S1. Growth of non-transformed and GPX-silenced rice lines at the reproductive stage.
